# Use of simulation for teaching biomedical sciences to undergraduate medical students- a scoping review

**DOI:** 10.1186/s12909-025-07819-y

**Published:** 2025-09-25

**Authors:** Joshua O. Owolabi, Katresa Gardner, Rasheed Agboola, Rekha R. Yesudas, Jennifer H. Shaw

**Affiliations:** 1 Department of Biomedical Sciences, Philadelphia College of Osteopathic Medicine, Moultrie, GA USA; 2 Academic Library, Philadelphia College of Osteopathic Medicine, Moultrie, GA USA

**Keywords:** Innovation, Medical education, SIM-based medical education, Pedagogy, Simulation

## Abstract

**Introduction:**

The objective of this scoping review was to understand the methods, extent and type of evidence in relation to the use of simulation or SIM-based medical education pedagogies to teach biomedical sciences to undergraduate medical students in accredited medical schools, globally. The review considers literature published between 2014 and 2024.

**Inclusion criteria:**

The review only considered reports about populations of students enrolled in accredited undergraduate medical education programs that train medical doctors, including DO/MD/MBBS/MBChB or equivalents in any country. Simulation, as a concept, is considered as reportedly used in the context of teaching knowledge, skills, or practice-related attitudes in biomedical science disciplines. Articles published in the English Language were considered.

**Methods:**

An initial search of the Cochrane and the Joanna Briggs Institute’s [JBI] Evidence-based Practice databases in October 2023 found no similar review. For this review, the primary databases searched included PubMed, ERIC, and Google Scholar. The JBI [Joanna Briggs Institute] SUMARI was the platform for screening, approval, extraction, synthesis, and review. For screening and appraisal, two members of the review team were required to approve an article.

**Results:**

A total of 18 articles were considered for this review out of the initial yield of 2,671. These included: 4 Analytical Cross-Sectional Studies- 2 from Australia, 1 from the USA, and 1 from China; 2 Systematic Review and Research Syntheses; 3 Randomized Controlled Trials- 1 from China and 2 from Italy; 6 Quasi-Experimental Studies- 1 from Taiwan, 1 from the Netherlands, 1 from the both China and United Kingdom, 1 from Sweden, 1 from Indonesia and 1 from the United States; and 3 Text and Opinion Studies. Findings highlight the growing use of simulation and technology-enhanced learning in medical education, improving competency, retention, and engagement. Simulation, aided by VR, AR, and PBL, enhances motivation and skills but cannot fully replace hands-on training. Limitations include inconsistent assessment impacts, cost challenges, and accessibility concerns.

**Conclusion:**

Simulation and technology-enhanced learning improve engagement, skills, and retention in medical education. Integration with traditional methods maximizes effectiveness. Virtual simulations aided by technologies, VR or AR offer immersive experiences but require careful implementation.

## Introduction

Simulation, especially in the context of medical education, represents a pedagogical approach that places learners within carefully constructed environments designed to model authentic real-world situations. Through these simulated contexts, students develop knowledge, competencies, and professional attitudes via direct engagement, observational learning, exploratory activities, and hands-on practice. Educational simulation has been characterized as a synthetic recreation of authentic processes or situations, specifically designed to facilitate learning through experiential engagement [1]. Within medical education, this approach has evolved into simulation-based medical education (SBME), which encompasses instructional activities that utilise simulation tools and technologies to recreate clinical environments [1] while addressing concepts, phenomena, and procedures central to medical training.

The adoption of simulation methodologies in medical education continues to expand, with documented achievements particularly evident in clinical skills training and the presentation of complex procedures and phenomena [2]; [3]; [4]. Given the heterogeneous nature of existing literature- spanning varied contexts, student populations, educational experiences, and measured outcomes—a comprehensive evaluation and synthesis of available evidence becomes essential for developing evidence-based practices and institutional policies. Furthermore, the pedagogical transformations prompted by the COVID-19 pandemic [5] have accelerated simulation adoption, either as alternatives to traditional methods or as complementary educational tools [6]; [7]. This review considers not only the trajectory and innovations in simulation-based learning throughout the past decade within our educational context, but also investigates recent transformations in practice, considering especially the year 2020’s significant global disruption due to the COVID-19 pandemic. Simulation methodologies are categorized according to multiple dimensions: fidelity (reflecting realism, complexity, and proximity to actual scenarios), technology (indicating the level of technological integration and advancement), and purpose (defining intended learning objectives and outcomes).

The application of simulation for teaching biomedical science principles, phenomena, and competencies constitutes a central focus of this review. Currently, no comprehensive review has examined recent developments and evolutionary changes in simulation’s role within biomedical science education in medical schools. This gap highlights the importance of the present study and its potential value to educational stakeholders by documenting simulation advances and their influence on educational practices and institutional culture. A scoping review methodology was considered quite appropriate given the absence of prior comprehensive reviews examining simulation applications in biomedical science education within medical training contexts. This review, therefore, sought to map current practices, trends, and scenarios in simulation implementation rather than merely quantifying and comparing outcomes against established benchmarks or standard practices. Publications meeting established inclusion criteria following systematic screening and evaluation were incorporated into the evidence synthesis process.

Despite simulation’s growing popularity, the field requires comprehensive guidance and frameworks for optimal implementation. The Sim Zone framework developed by Boston Children’s Hospital represents a significant contribution toward establishing standards and best practices in medical simulation [8]. Contemporary simulation applications span three primary domains: knowledge-focused instruction emphasizing cognitive development, skills-based training targeting psychomotor competencies (which represents the most prevalent application), and attitude-centered education promoting collaborative teamwork and positive interprofessional relationships through affective learning.

Simulation in medical education is often typically associated with clinical and surgical skills laboratories and active learning environments. Nevertheless, recent initiatives have expanded its application beyond skills training to encompass knowledge transmission and affective domain competencies. Educational experiences have demonstrated simulation’s effectiveness in teaching topics in Anatomy, Physiology, Pharmacology, and related disciplines within integrated curricula. These applications focus on conveying scientific and clinical knowledge while fostering collaborative teamwork. Medical educators have documented their experiences, outcomes, and educational impacts when using simulation to address specific disciplinary topics and concepts [9]; [10]. Efforts are also being made to promote integrated and comprehensive simulation-based medical education rather than siloed skill- or knowledge-based simulation activities.

While preparing for this scoping review, a preliminary literature search was conducted in 2023 across MEDLINE, the Cochrane Database of Systematic Reviews, and JBI Evidence Synthesis. No existing or ongoing systematic or scoping reviews specifically addressed this topic. Therefore, this scoping review was aimed at evaluating the breadth of available literature regarding simulation applications in teaching biomedical science disciplines—including but not limited to Anatomy, Biochemistry, Microbiology, Pathology, and Physiology- within medical education curricula for medical students, globally [9]; [11].

### Review question

In what ways has simulation or SIM-Based Medical Education been used to teach biomedical sciences to undergraduate medical students [DO/MD/MBBS/MBChB] in the past decade i.e. 2014–2024?

### Inclusion criteria

#### Participants

These included undergraduate DO/MD/MBBS/MBChB students. Studies on the use of simulation to teach undergraduate medical students were conducted. The disciplines to be considered were those that are indicated in the study and/or program curriculum as biomedical or basic medical science disciplines. Articles or publications to be considered are original studies and reports. Excluded from the reviews were comments, commentaries, opinions, and other publications that are not peer-reviewed or those that are not obtained from education research or reports.

#### Concept

This was centred on simulation or SIM-based medical education. The primary concept of interest is simulation or SIM-based medical education for undergraduate medical education. Only the reported use of simulations for biomedical or basic medical science disciplines was considered. Articles reporting the use of simulations for clinical or critical skills or for teaching concepts and phenomena in disciplines not categorized as biomedical sciences were excluded. Also, the reported use of simulation for special skill acquisition purposes not considered to be a component of biomedical sciences in undergraduate medical education was excluded.

#### Context

Biomedical science disciplines in undergraduate medical education, globally. By virtue of context, the review considered reports or publications from any accredited institution, school or college considered an orthodox medical school that trains students to become medical doctors. These include osteopathic schools that confer the doctor osteopathic medicine (DO) degree on graduates as well as allopathic schools that confer medical doctor (MD) bachelor of medicine and bachelor of surgery (MBBS or MBChB), degree. Qualified articles from any country of the world were considered. Commercial articles and non-peer-reviewed publications such as special reports, were excluded (See appendix 1).

### Types of sources

This scoping review considered both experimental and quasi-experimental study designs, including randomized controlled trials, non-randomized controlled trials, before and after studies, and interrupted time-series studies. Also, analytical observational studies, including prospective and retrospective cohort studies, case-control studies, and analytical cross-sectional studies were considered for inclusion. The review also considered, for inclusion, descriptive observational studies by designs, including case series, individual case reports, and descriptive cross-sectional studies. Furthermore, qualitative studies that focus on qualitative data including, but not limited to, designs such as phenomenology, grounded theory, ethnography, qualitative description, action research and feminist research were considered. In addition, systematic reviews that meet the inclusion criteria were also considered, based on the research question. Commentaries and case reports were also considered for inclusion in this scoping review.

## Methods

The scoping review was conducted in accordance with the JBI methodology for scoping reviews [12].

### Search strategy

The search strategy aimed to locate both published articles in mainstream databases and grey literature. A three-step search strategy was utilized in this review. First, there was an initial limited search of MEDLINE (PubMed) and CINAHL (EBSCO). Thereafter, text words in the titles and abstracts of relevant articles and the index terms were used to describe the articles were used to develop a complete search strategy for the databases indicated (see Appendix #1). The search strategy, including all identified keywords and index terms, was then adapted for each included database and information source. The reference list of all included sources of evidence such as original studies, reports, and reviews, was screened for additional studies. Only studies published in the English language were included since reviewers were primarily English speakers without the same level of proficiency in other languages. Studies published between the 2014–2024 period were considered. Additional databases explored include BMC Medical Education, ERIC, OVID, and Google Scholar to identify literature on this topic. Grey literature sources including ProQuest Dissertations and Open Access Theses and Dissertations (OATD) were also explored and considered.

### Study/Source of evidence selection

**Fig. 1:**
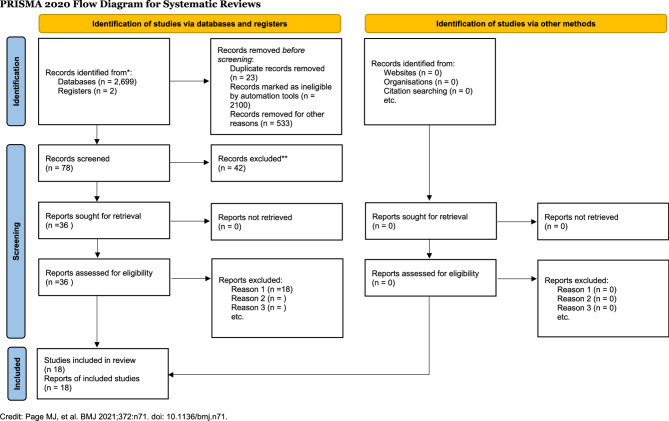
PRISMA flow diagram for the scoping review showing the sources, initial yields of search and the process of getting the final 18 articles that met the inclusion criteria for the review

Following the search, all identified citations were collated and uploaded into JBI SUMARI; duplicates were removed. Titles and abstracts were screened by at least two independent reviewers for proper assessment against the inclusion criteria for the review. Potentially relevant sources were retrieved in full, and their citation details were imported into the JBI System for the Unified Management, Assessment, and Review of Information- JBI SUMARI, by the Joanna Briggs Institute, Adelaide, Australia [13]. The full text of selected citations was read and reviewed in detail against the inclusion criteria by two or more independent reviewers. Reasons for the exclusion of sources of evidence in full text that do not meet the inclusion criteria were recorded and reported in the scoping review. Any disagreements between the reviewers at each stage of the selection process were resolved through quality discussions and consideration for the inclusion versus exclusion criteria, or with an additional reviewer/s. The results of the search and the study inclusion process are reported in full in the final scoping review and presented in a PRISMA i.e. Preferred Reporting Items for Systematic Reviews and Meta-Analyses, flow diagram [14]. (See Fig. 1).

#### Data extraction

Following the JBI protocol for scoping review, and with the use of the JBI SUMARI platform [12];[13], data was extracted from papers that met inclusion criteria, hence, included in the scoping review by two or more independent reviewers using the data extraction tool developed by the reviewers [12]. Extracted data includes specific details about the participants, concept, context, study methods, and key findings relevant to the review question/s. The data extraction tool template was adapted and used during entire the process of extracting data from each included evidence source. Modifications were detailed in the scoping review. Any disagreements that arise between the reviewers were resolved through discussion, or with an additional reviewer/s.

## Data analysis and presentation

Following extraction, a narration of evidence synthesis was presented. in alignment with the review objective and question(s). As much as possible, data were presented graphically or in diagrammatic or tabular form. In each instance, a narrative summary will accompany the tabulated and/or charted results and will describe how the results relate to the review objective and question/s.

## Results

### General description of selected articles

In total, 18 articles were considered and included in this review for final analysis and synthesis of evidence out of the initial yield of 2,699 [2,699 from databases; and 2 from registers] [See Table 1]. These included: Four (*n* = 4) Analytical Cross-Sectional Studies with 2 from Australia, 1 from the USA, and 1 from China; two (*n* = 2) Systematic Review and Research Syntheses; three (*n* = 3) Randomized Controlled Trials including 1 from China, and 2 from Italy; six (*n* = 6) Quasi-Experimental Studies including 1 from Taiwan, 1 from the Netherlands, 1 from both China and United Kingdom, 1 from Sweden, 1 from Indonesia, 1 from the United States; as well as three (*n* = 3) Text and Opinion articles.

**Table 1:** Characteristics of included studies analytical Cross-Sectional study

Study	Country	Setting/context	Participant characteristics	Groups	Outcomes measured	Main description of results		
1. Maurizio Costabile. 2020[16].	Australia	Laboratory biochemistry session for combined undergraduate STEM and medical science students. Covered key aspects of laboratory practice, including fundamental mathematical skills, reading, and setting a pipette, basic Biochemistry assays, protein quantification, and enzyme kinetics.	Undergraduate students enrolled in STEM and medical science courses in an Australian university.	Laboratory biochemistry session for combined undergraduate STEM and medical science students.	Laboratory assessment scores	The online simulations used to teach biochemistry laboratory content were well received and found acceptable by STEM and medical science students. There were no significant changes in laboratory assessment scores following the interventions.		
2. Jackson J, Stacey R, Korczyk S, Williams D. 2020[15].	USA	SP for simulation-based medical education was issued in the context of a Simulated Cardiology Clinic in an effort to teach basic clinical concepts in cardiovascular cases to Year 2 medical students. Cardio-Vascular Medicine course directors met with students for a large-group debriefing by highlighting the key learning points.	Clinic activity for second-year medical students consisting of standardized patient (SP) cases for cardiovascular (CV) diseases	A class of second-year medical students in the pre-clinical phase of undergraduate medical education	Application of scientific skills to solving cardiovascular cases and acquisition of basic level of diagnostic skills for differential diagnosis	Standardized patient encounters representing cardiovascular conditions can effectively provide suitable chances for students to integrate basic science knowledge and clinical skills.		
3. Chen H, Kelly M, Hayes C, van Reyk D, Herok G. 2016[17].	Australia	Assessment performances improved in measurable ways; students indicated satisfaction. Lecture content is reinforced through practical learning experiences in clinical environments.	Biomedical undergraduate students using simulation for learning	Students enrolled in the biomedical courses that were foundational to the clinical phase of medical education.	Learners’ performances in formal course assessments	Clinical simulation to deliver case studies was more effective for learning pathophysiology concepts than traditional case study delivery methods. Results, following the use of simulation, Indicated a significant shift from a pass grade (50–64) toward higher grades and mark ranges (75–84 and 85) when we compared time period of 2010 with 2011–2013.		
4. Yu L, Wang W, Liu Z, Liu Z, Xu Y, Lin Y. 2023[27].	China	1 st year medical students in experimental lab skills course in China	Medical students in year 1 of medical school in China		Quality of learning experience and learners’ confidence afterwards.	Results showed evidence of familiarity and confidence gained as well as theoretical genetic understanding, but cannot replace experimental learning entirely.		
Study	Review objectives	Descriptions of interventions/phenomena of interest	Descriptions of outcomes included in the review	Descriptions of contexts included in the review	Search details	Number of studies and participants included	Appraisal instruments used	Description of main results
5. Abhishek Nagarajappa, Simran Kaur. 2024[18].	The use of simulation-based medical education for undergraduate and graduate medical education, particularly on anesthesiology to first year medical students; included 41 studies.	The use of Simulation-Based Medical Education (SBME)- for undergraduate and graduate medical education in different contexts and programs is generally applicable to undergraduate medical education. It has been specifically mentioned in programs such as anesthesiology and cardiopulmonary resuscitation (CPR) training within clinical education. SBME provides a hands-on, experiential learning environment that helps bridge the gap between theoretical knowledge and real-world clinical practice, allowing trainees to develop and refine their skills in a controlled and safe setting.	Outcomes considered in the review included improvements in medical education assessment outcomes and improvements in the communication skills of undergraduate medical students.	Use of SBME for both undergraduate and graduate medical education; For undergraduate medical education at a preclinical phase SIM-based medical education was reportedly perceived by first-year medical undergraduate students to better enhance their communication skills with actual patients compared to simulated patients based on focus group discussion.	A search strategy was developed and adapted for searches on multiple databases including PubMed, Medline, and Scopus	41 studies	Search results on the subject of interest, and based on the search strategy were synthesized by a qualitative method.	General improvement in medical education outcomes; improved communication skills in undergraduate medical students. Other aspects addressed undergraduate clinical education. Study also revealed that including simulation in curriculum helps to benefit in skill enhancement and shortening the student learning curve.
6. Santos VA, Barreira MP, Saad KR. 2022[20].	The reviews objective was to consider the the technological means for teaching and learning human anatomy as it applied to medical courses over a ten-year period with the aim of identifying the major technologies and their reported educational impacts.	Use of technologies and technology-enabled resources (3-D printing, extended reality, digital tools) and platforms, and resources for teaching human anatomy in medicine.	Impact of technology on student learning and educational quality of learning experience as measured by test-related performance, acceptance, and overall implementation success.	Review of the learning and training experiences of students enrolled in medical programs taking human anatomy courses were included in a review of 102 studies from five continents: Asia, Australia, Europe, North America, and South America.	Using a standard search strategy, search was conducted in each of MEDLINE, Scopus, ERIC, LILACS, and SciELO databases yielding an initial result of 875 identified articles, from which 102 were included in the eventual analysis.	102 studies were included	Preferred reporting items for systematic re-views and meta-analyses (PRISMA) guideline	From the 102 studies included, several technological resources for teaching anatomy in the context of medicine were identified and categorized as follows: three-dimensional printing, extended reality (including AR and VR), and digital tools, with simulation also indicated. Overall, the review showed neutral or beneficial results in the utilization of educational technology and resources when integrated with traditional teaching methods. Nevertheless, the review showed that among the edtech products considered, internet-supported technologies and 3D printing are the most acceptable on the basis of student learning and cost-benefit analysis.
Study	Country	Setting/context	Participant characteristics	Groups	Outcomes measured	Description of main results		
7. Hongxiang Xie LW. 2022[29].	China	Randomly selected students from 2018 class enrolled in offline LBL (lecture based learning) and traditional experimental teaching methods	Pre-clerkship medical students in laboratory class (clinical biochemistry) in China	1. Randomly selected students from 2018 class enrolled in offline LBL (lecture based learning)2. Students from 2018 class enrolled traditional experimental teaching methods	Assessment/examination scores in theoretical knowledge; and experimental operational skills.	PBL and virtual SIM lab improved examination scores in theoretical knowledge and experimental operational skills (p=0.0095) relative to LBL and traditional SIM lab.		
8. Cercenelli L, De Stefano A, Billi AM, Ruggeri A, Marcelli E, Marchetti C, et al. 2022[28].	Italy	A Pilot study of an innovative educational tool combining AR Technology and 3D Printing i.e. AR+3D printing model (AEducaAR) versus traditional atlas learning.	62 2nd year medical students, selection criteria -volunteered to participate then randomly assigned groups	1. Group exposed to augmented reality (AR) technology2. Group exposed to 3D printed model for topographical learning	Standard course assessments i.e MCQ exam and practical; and engagement/interest/motivation through feedback survey/questionnaire.	There was no statistical (t test, unpaired) difference in objective test results (MCQ exam and practical), however increased engagement/interest/motivation indicated on Likert scale feedback survey/questionnaire (43/62 response rate)		
9. Arcoraci V, Squadrito F, Altavilla D, Bitto A, Minutoli L, Penna O, et al. 2019[30].	Italy	Medical education: Basic life support task trainer, versus full body simulation training unit (low fidelity), versus full body ALS simulator high fidelity group.	Parallel, randomized 5th year medical students; 90 out of 225 students participated in the study. After a lecture to all, students were randomly assigned to 3 groups (sham, low fidelity, high fidelity) i.e. 30 students per group.	3 groups: sham, low fidelity, and high-fidelity simulation exposure groups	Pre- and post exposure assessment of competences.	20 MCQ pharma assessment was administered at baseline, after lecture, and immediately after SIM (simulation) and 3 months later (students blinded from results). Non-parametric approach was used- Chi-squared test and Kruska-Wallis test for independent samples to compare 3 groups; male versus female participants were compared using Mann-Whitney U test. High fidelity SIM significantly increased correct answers (p<0.001); no difference between sham and low fidelity; there was a statistically significant decline in retention for sham and low fidelity but not for high fidelity.		
Study	Country	Setting/context	Participant characteristics	Groups	Outcomes measured	Main description of results		
10. Syed Abdul S, Upadhyay U, Salcedo D, Lin C-W. 2022[25].	Taiwan	Use of virtual reality to learn sonography in medical education context. Students could learn 3D Anatomy, cut and rotate 3-D animated image and learn better than with 2D images. Virtual reality was used to teach anatomy, physiology, and other concepts related to medical education and practice. Emphasis included: Emergency room (ER) experience to resuscitate patients; VR anatomy to enhance sonography learning; and thoracentesis VR training.	Use of VR for virtual simulation of pre-clinical anatomy and physiology for junior medical students in Taiwan.	Learners'experience and satisfaction as self-reported.	Virtual reality simulations satisfactorily presented anatomy, physiology and related medical education concepts to undergraduate medical students. Authors advocated for an urgent need for the collaboration of medical institutes and technology industries on developing education-related VR content and simulations as an alternative option for traditional training and teaching.	Impact and applications of virtual reality (VR) in medical education and clinical practice; evaluating VR’s effectiveness in enhancing medical training through immersive simulations, anatomy learning, emergency room experiences, and thoracentesis procedures.		
11. Bölek KA, De Jong G, Van der Zee CEEM, Cappellen van Walsum A-M, Henssen DJHA. 2022[21].	Netherlands	Neuroanatomy education in a structured setting where students used AR in combination with anatomical specimens in dissection rooms during practical sessions.	Second-year undergraduate medical and biomedical sciences students (n = 222; mean age: 19.7) at Radboud University Medical Center, Nijmegen, the Netherlands.	Students'motivation to study neuroanatomy.	The study considered the use of augmented reality (AR) tool, GreyMapp-AR, for learning neuroanatomy alongside traditional dissection room sessions and prosected specimens.	Study found that GreyMapp-AR increased student motivation to study neuroanatomy, particularly in understanding subcortical structures. Biomedical sciences students reported higher confidence using AR, while male students rated AR as more relevant than females		
12. Monteiro O, Bhaskar A, Ng AKM, Murdoch CE, Baptista-Hon DT. 2021[22].	China; United Kingdom.	Undergraduate medical students'using of the LabHEART software as a virtual simulation of heart EC-coupling related activities; students simulate and measure action potentials, intracellular calcium changes, and cardiomyocyte contraction.	Undergraduate medical, biological sciences, and pharmacology students.	Learning outcomes as indicated in the curriculum or program document.	LabHEART simulation satisfactorily replaced animal heart experimentation and any other learning model that was previously used for heart physiology practical/hands-on sessions.	LabHEART simulations enhanced active learning by allowing students to explore cardiac physiology, ion channel modifications, and pharmacological effects, improving conceptual understanding in various educational formats​.		
13. Ohlsson L, Moreira A, Bäck S, Lantz J, Carlhäll C-J, Persson A, et al. 2023[23].	Sweden	Undergraduate medical education year 1 course on heart anatomy and physiology	From a total of 110 Medical students enrolled in a medical school in Sweden, 97 (88%) participated in the voluntary lecture'Immersion of the anatomy and physiology of the heart by four-dimensional visualization.	Impacts of the reported use of a 4D model i.e incorporated 3D anatomy with blood flow simulation and heart-beating actions in a virtual simulation context on learning as indicated by summative assessments performances.	Summative assessments indicated a significantly improved mean score (18.1 ± 4.5 vs 20.3 ± 4.9, p = 0.002); partly attributed to enhanced knowledge of myocardial hypertrophy and pressure–velocity differences over a stenotic valve. Students'self-reported data also indicated acceptance of the method and satisfaction.	Study found that 4D visualization of cardiac anatomy and blood flow significantly improved students'understanding of cardiac physiology, particularly in myocardial hypertrophy and stenotic valve dynamics​		
14. Soraya GV, Astari DE, Natzir R, Yustisia I, Kadir S, Hardjo M, et al. 2022[26].	Indonesia	first-year medical students (whole class)	Year 1 medical students virtual lab for blood typing taking antibody and blood biochemistry theoretical lectures delivered via zoom; recruited into a study that considered the implementation of virtual laboratory simulations (vLABs) for medical biochemistry.	Post-intervention course assessments; learners motivation.	Statistically significant increase in scores (p<0.0001 Wilcoxon signed rank test) on post intervention assessment was observed, also between first and best attempt scores; cognitive and motivational benefits were also reportd via surveys, while there were also noted technical (non-direct interaction with equipment) and language (non-native language) barriers. Also, virtual lab efficacy was assessed but not compared to in-person lab.	Study showed that virtual laboratories significantly improved medical biochemistry students'understanding, motivation, and performance, despite challenges like language barriers and technical difficulties​.		
15. Sheakley ML, Gilbert GE, Leighton K, Hall M, Callender D, Pederson D. 2016[19].	United States	2009 cohort (pre intervention) v 2011 cohort/class of medical students (post intervention)	Undergraduate medical students in the US enrolled in sessions involving lecture plus simulation for cardiac anatomy, and heart sounds.	2 cohorts: 2009 cohort (pre intervention) v 2011 cohort/class of medical students (post intervention)	Written exam scores	Written exam scores increased, as well as short term understanding; also, predictive score for summative exam passage statistically increased.		
Study	Type of text	Population represented	Topic of interest	Setting/context/culture	Stated allegiance/position	Description of main argument(s)		
16. Moro C, Gregory S. 2019[50].	Text and opinion: Chapter in a special series	Anatomy and physiology educator and learners in the context of medical education; use of educational technology to support off-campus anatomy physiology learning in medicine and biomedical sciences.	Technologies that help to with Visualizations of anatomy and physiology structures and concepts including a range of visualizations utilized are like 3D-printing, simulated dissections, holograms, simulation tables, virtual and augmented reality, smart tablets and touch screen devices..	Undergraduate medical education.	Lecture content is reinforced through self-directed technological learning resources in off-campus study.	Technology utilized outside classrooms and laboratories to reinforce lecture content is more effective to learn Human Anatomy and Physiology concepts than traditional learning methods including textbooks and lecture notes and recordings.		
17. Arjomandi Rad A, Subbiah Ponniah H, Shah V, Nanchahal S, Vardanyan R, Miller G, et al. 2023[24].	Chapter in a Series/Text	Undergraduate and graduate medical trainees; also professionals benefiting from supplementary resources for anatomy teaching and improved clinical experiences.	Extended Reality (XR) as a supplement to cadaver-based teaching. XR (Extended Reality) use in medical education and additional useful resources with interoperable value in surgery.	the use of Extended Reality (XR) technologies, including Virtual Reality (VR), Augmented Medical students studying anatomy in low-middle income countries; post grad training in surgery (authors in UK). Use of Extended Reality XR, Augmented Reality (AR), and Mixed Reality (MR) in medical education, with an emphasis on Anatomy and Surgery. These technologies aim to improve anatomy teaching, surgical training, and interpersonal skills development through immersive, interactive, and risk-free educational experiences.	The comparison is between XR technologies and traditional medical education methods, such as cadaveric dissection for anatomy learning and hands-on surgical training with real patients. Traditional methods are often costly, less accessible, and carry risks to patient safety, which XR seeks to address through safer, more accessible simulation-based learning.	XR has proven to be most relevant when typically applied to Anatomy and Surgery, offering benefits such as superimposing digital and interactive virtual dimensions onto the real world, with both interacting without disruptive interference. The use of XR in medical education has demonstrated improved learning outcomes, including a better understanding of anatomy, enhanced surgical skills, and reduced educational costs over time. XR also promotes accessibility to quality education in LMICs, bridging the gap caused by limited resources and experienced mentors.		
18. Sá-Couto C, Patrão L, Maio-Matos F, Pêgo JM. 2016[31].	Narrative Review and Expert Opinions	Medical students in Portugal	Biomedical Simulation: Evolution, Concepts, Challenges and Future	Biomedical simulation for medical education with an emphasis on Portugal.	Simulation has become an important and integral aspect of medical education; in the context of Portuguese medical education, formal integration of simulation in healthcare sciences education has increased in the last years, as a result of curricular reforms.	Integrating simulation-based training into healthcare curricula is important, albeit, challenging. Basic concepts and considerations were highlighted to include: Tools, scenario design, actual scenario, debriefing and feedback and assessments. Integration of simulation into medical education and practice was a main point of advocacy.		

Most articles that meet the inclusion criteria are Quasi-Experimental Studies (30%). Altogether, most articles meet scholarly rigor that could make analyses and subsequent inferences reliable based on the strength of evidence. In terms of representativeness by global regions, most articles are from developed or otherwise high-income countries, also known as the Global North. Only Taiwan and China have articles from authors in Asia, whereas no article was found—considering the premise and purpose of the study, as well as the inclusion and exclusion criteria- from Africa, South America, the Caribbean, and the Middle East, in the final article selection. In terms of timeline, the articles considered were published between 2016 and 2024 representing the up-to-date nature of the search, hence the eventual evidence. Data and empirical findings in these publications were first extracted, organized and presented in Table 1; then, analysed and synthetized following the JBI methods and presented in Table 2 [12]; [13]; [14].

**Table 2:** Major themes emerging from the use of simulation for teaching biomedical sciences to medical students in the context of this review

SN	Key Considerations	Main Theme and Sub-theme from Articles
1	Simulation Enhanced Knowledge and Skill Acquisition	1. Simulation Enhanced Knowledge and Skill Acquisition in Medical Education o Simulation improved biochemistry lab learning but not assessment scores [15]. o Simulated standardized patients helped enhance cardiovascular clinical skills in pre-clinical students [16]. o Clinical simulations enhanced learning of pathophysiology concepts [17]. o Simulation improved medical skills and shortens learning curves [18]. o Lecture plus simulation improved cardiac anatomy understanding [19].
2	Simulation Enhanced Learners Experience in Anatomy and Physiology	2. Technology-Enhanced Learning in Anatomy and Physiology o Educational technology aided human anatomy teaching with neutral or positive impact [20]. o Simulated concepts via augmented reality boosted motivation in neuroanatomy education [21]. o Virtual simulation practically replaced animal models for heart physiology learning [22]. o 4D visualization improved heart anatomy and physiology learning outcomes [23]. o Extended reality supported anatomy and surgical training [24].
3	Virtual Simulations Forms	3. Effectiveness of Virtual and Augmented Reality in Medical Training o Virtual reality enhanced sonography learning and emergency medicine training [25]. o Virtual lab simulations improved blood typing knowledge and motivation [26]. o Virtual labs helped with confidence but could not replace experimental learning [27]. o Augmented reality increased student engagement but not test performance [28].
4	Simulation Improved Competency and Retention	4. Simulation and Problem-Based Learning Improved Competency and Retention o PBL and virtual simulation improved theoretical and practical skills [29]. o High-fidelity simulation improved pharmacology learning and retention [30].
5	Integration of Simulation into the Curriculum	5. Integration of Simulation-Based Training into Medical Curricula o Simulation integration into medical curricula considered to be increasingly popular [31].

#### Simulation enhanced knowledge and skill acquisition in medical education

Evidence shows that simulation-based learning has been widely adopted to improve knowledge acquisition and practical skill development in medical education. Simulation of clinical presentation in the form of standardized patients has been reported to enhance cardiovascular clinical skills in pre-clinical students, thereby facilitating the application of basic science knowledge to real-world clinical encounters [15]. In another instance biochemistry laboratory sessions were facilitated with simulations. These simulations were well received by students, although no significant improvements in assessment scores were observed [16]. Also, clinical simulations were found to enhance the learning of pathophysiology concepts, leading to improved performance in assessments [17]. Generally, simulation-based education has been shown to improve medical students’ skills, reducing the learning curve and better preparing them for clinical practice [18]. In addition, integrating lecture-based teaching with simulation has been linked to improved cardiac anatomy understanding, reinforcing theoretical concepts through experiential learning [19].

#### Simulation is a major form of technology-enhanced learning in anatomy and physiology

Advancements in educational technology have significantly influenced anatomy and physiology education. Various forms of simulation- based on modality and fidelity- are used to enhance learners’ experiences in Anatomy and physiology. The integration of digital tools such as three-dimensional printing, virtual reality, and augmented reality has been found to support student learning with generally neutral or positive impacts on educational outcomes [20]. In neuroanatomy education, the use of augmented reality has been shown to enhance student motivation, particularly in understanding complex structures such as subcortical regions [21]. Virtual simulations have also been successfully used to replace traditional animal models in heart physiology learning, providing a more ethical and interactive learning experience [22]. Moreover, the implementation of four-dimensional visualization techniques in teaching heart anatomy and physiology has led to significantly improved student performance in summative assessments [23]. Extended reality technologies have also emerged as valuable tools in surgical and anatomy training, offering immersive and interactive experiences that enhance both theoretical understanding and practical application [24].

#### Effectiveness of virtual and augmented reality in biomedical science teaching

Virtual and augmented reality have demonstrated effectiveness in various aspects of medical education, with an emphasis on biomedical sciences, though with some limitations. In these instances, concepts, phenomena, or procedures are simulated. They have been used as avenues to present applied knowledge and clinical correlations. For example, virtual reality-based training has been particularly beneficial in enhancing sonography education, emergency medicine training, and procedural learning by providing interactive and immersive experiences [25]. In medical biochemistry, virtual laboratory simulations have been shown to improve students’ understanding of blood typing, with significant gains in knowledge and motivation [26]. However, while virtual labs reportedly contribute to increased familiarity and confidence, they did not satisfactorily replace hands-on experimental learning [27]. Similarly, augmented reality has been found to increase student engagement in learning anatomical structures, though it has not consistently translated into significant improvements in test performance [28]. These findings highlight the importance of integrating virtual and augmented reality as supplementary tools rather than full replacements for traditional learning methods. The principal approach has been to simulate concepts, phenomena, procedures, or experiences.

#### Simulation and problem-based learning improved competency and retention

Simulation-based learning, in combination with problem-based learning (PBL), has been found to significantly enhance competency and knowledge retention among medical students. Studies show that integrating PBL with virtual simulation improved both theoretical knowledge and practical skill acquisition, particularly in laboratory-based subjects such as clinical biochemistry [29]. High-fidelity simulation training has also been demonstrated to improve pharmacology learning and long-term retention of concepts, as students in high-fidelity simulation groups performed better on assessments compared to those in low-fidelity or traditional learning environments [30]. These findings support the use of simulation as an effective pedagogical tool for fostering deeper learning and ensuring knowledge retention in medical education.

#### Integration of simulation-based training into medical urricula

The integration of simulation-based training into medical curricula has gained increasing recognition as an essential component of modern medical education. Over the years, there has been a growing emphasis on incorporating simulation into healthcare science curricula, leading to its formal adoption in many medical education programs [31]. Simulation-based training is now widely used to enhance the learning experience, providing students with opportunities to practice clinical skills in a safe and controlled environment before engaging in real patient care. This shift reflects the increasing acknowledgment of simulation as a critical tool for improving competency, confidence, and preparedness among medical trainees.

## Discussion

### An overall theme for this review

*An overall theme for this review could be stated as "Simulation and Technology-Enhanced Learning is Transforming Medical Education and Skill Acquisition." *The integration of simulation-based and technology-enhanced learning in medical education has gained widespread recognition for its ability to enhance knowledge acquisition, clinical skills, and student engagement. The findings of this systematic review demonstrate that simulation contributes to improved competency in biomedical sciences education (in the context of medical education) by providing experiential learning opportunities, reinforcing theoretical concepts, and shortening the learning curve for students. Despite its numerous benefits, simulation-based learning has some limitations, particularly when used as a complete replacement for traditional laboratory or clinical experiences. This discussion explores the impact of simulation and technology-enhanced learning across five key thematic areas: (1) simulation’s role in skill acquisition, (2) simulation and technology-enhanced anatomy and physiology education, (3) virtual and augmented reality in medical training, (4) problem-based learning (PBL) and competency development, and (5) the integration of simulation into medical curricula.

### Simulation enhances knowledge and skill acquisition in medical education

Simulation has been widely acknowledged as a valuable tool for developing clinical and scientific competencies in medical and biomedical sciences education. The use of standardized patients, virtual laboratories, and simulated clinical encounters allows students to apply theoretical knowledge in realistic but controlled environments, thus bridging the gap between theory and practice [32]. For example, standardized patient (SP) encounters have been shown to enhance cardiovascular clinical skills in pre-clinical students, improving their ability to integrate basic science knowledge with diagnostic reasoning [15]. Similarly, clinical simulation has been found to enhance students’ understanding of pathophysiology, leading to improved assessment outcomes [17].

However, the effectiveness of simulation is highly dependent on its implementation. In biochemistry laboratory education, for instance, students responded positively to simulation-based interventions, but no significant improvement in assessment scores was observed [16]. This suggests that while simulation can enhance student engagement and motivation, it may not always lead to direct improvements in summative assessments, highlighting the need for strategic integration with other instructional methods. Studies have also indicated that repeated exposure to simulation-based training helps to reinforce learning, reducing cognitive overload and improving long-term retention [33].

With the increasing popularity of integrated curriculum in medical education globally, simulation is proving to be a means of incorporating clinical correlation and the understanding of pathophysiology into the pre-clinical or pre-clerkship phase of medical education. Noting that this phase is also predominantly the basic medical or biomedical education phase of medical education, it provides a significant opportunity to diversify pedagogical practice, enhance learners’ appreciation for pathological and clinical concepts, and promote engagement with learning materials. Therefore, simulation-based medical education could be valuable to also mitigate over-dependence on didactics to present materials, especially for those that emphasize application knowledge and clinical correlations [34]; [35]; [36]. While a significant increase in assessment might not have been reported in the cases considered, increased engagement is still a significant gain as this could further promote self-efficacy and commitment to further study, which are aspects of medication that are important to professional development and a commitment to life-long learning. Very importantly, skill acquisition at the pre-clinical or pre-clerkship phase is an outstanding benefit that simulation based medical education could offer, especially ahead of the didactics.

### Technology-enhanced learning in anatomy and physiology

Technological advancements have played a significant role in transforming anatomy and physiology education. The use of digital tools such as three-dimensional printing, virtual dissection tables, and augmented reality (AR) applications has been shown to enhance students’ understanding of anatomical structures and physiological processes [20]. In particular, AR-based neuroanatomy education has been found to increase student motivation, especially when learning complex anatomical structures [21].

Virtual simulation tools have also been used as alternatives to traditional wet-lab experiments. For instance, the LabHEART software, which allows students to simulate cardiac electrophysiology experiments, has effectively replaced animal models in heart physiology education [22]. Additionally, four-dimensional visualization techniques have been successfully implemented in cardiovascular education, significantly improving students’ ability to understand myocardial function and hemodynamic changes [23].

It is not surprising that teaching anatomy and physiology to students in the biomedical phase of medical education significantly benefited from the use of simulation for teaching important topics and concepts. The approach in this instance also suggests that the structure and function of organs and systems were taught or presented in combination in an effort to not only integrate knowledge in the anatomy and physiology domains but also properly conceptualize the structural organizations that are responsible for functional dynamics. This approach is very significant as it could serve as an optimal way of learning anatomy and physiology in an integrated manner yet devoid of excessive cognitive load that might be placed on learners in attempts to teach this in combination using didactic approaches or other means. Simulation, therefore, is an important way to integrate knowledge across two or more domains of medical education while still effectively managing the cognitive load for learners. The fact that concepts, phenomena, and procedures can be visualized in simulation-based medical education also helps to present learning material in modalities that learners can readily perceive, conceptualize, and effectively retain or apply subsequently [37]. Anatomists and physiologists therefore collaborate to teach fundamental concepts especially those that are significantly integrated and that may somehow be difficult for learners to conceptualize individually on the basis of domain and so properly integrate in terms of structural and function combined, such as the anatomy and physiology of the human heart and certain functions of the nervous system.

Despite the aforementioned benefits, the effectiveness of simulation and its technologies is influenced by factors such as usability, student engagement, and accessibility. While digital tools generally provide a positive learning experience, their benefits are maximized when integrated with traditional methods rather than used in isolation [38]. Furthermore, the cost of implementing high-fidelity simulation and virtual reality-based learning can be a limiting factor for many institutions, particularly in low-resource settings [39].

### Effectiveness of virtual and augmented reality in medical training

It is worth mentioning that from the reports, virtual and augmented reality (VR/AR), was adapted for simulation-based medical education in the form of virtual modalities. VR and AR have gained traction in medical education due to their ability to create immersive and interactive learning environments. VR has been particularly effective in sonography education, emergency medicine training, and procedural skill development [25]. In biochemistry education, virtual laboratory simulations have improved students’ understanding of blood typing, leading to increased knowledge retention and motivation [26].

Despite these benefits, VR and AR are not without limitations. Available evidence has shown that while virtual labs increase student confidence and engagement, they could not fully replace hands-on experimental learning [27]. Similarly, AR applications have been found to improve student interest in learning anatomical structures but have not consistently led to significant improvements in test performance [28]. These findings suggest that while immersive technologies offer promising educational advantages, they should complement, rather than replace, traditional hands-on learning experiences.

Another key consideration is the potential for cognitive overload when using highly immersive VR/AR tools. A study suggested that excessive visual stimuli in VR environments can reduce learning efficiency if students are not adequately guided [40]. To mitigate this issue, instructors must carefully design VR-based curricula to ensure that the cognitive load remains manageable and that learning objectives are clearly defined. From these findings, it would be appropriate to state that virtual modalities of simulation are now being explored in the forms of VR and AR for the simulation of functions, concepts, phenomena, or even clinical procedures and activities. What this might further imply is that technological advancement is also contributing to the expansion and advancement of simulation based medical education both in terms of modalities and fidelity. Although virtual and augmented realities have served as learning resources in medical education and other aspects of higher education, their use specifically for simulation-based medical education as reported in this study, is worth being emphasized. It will also imply that resources in different modalities can be applied to simulation-based medical Education. Nevertheless, it is important to note that application of appropriate pedagogical frameworks and principles will be required to optimize the use of these virtual and augmented reality resources for optimal educational impact and learning outcomes [41]; [42].

### Simulation and problem-based learning improve competency and retention

The combination of simulation-based learning with problem-based learning (PBL) has been shown to enhance medical students’ ability to apply knowledge in real-world contexts. Studies indicate that PBL, when integrated with virtual simulation, significantly improves students’ theoretical understanding and practical skills, particularly in laboratory-based subjects [29]. High-fidelity simulation has also been demonstrated to enhance pharmacology education, improving both short-term knowledge retention and long-term competency development [30]. These findings align with broader research on PBL, which suggests that active learning strategies, such as case-based discussions and simulation exercises, lead to deeper understanding and better retention of complex medical concepts [43].

However, the effectiveness of PBL and simulation depends on several factors, including facilitator expertise, student engagement, and curriculum design. It has also been shown that poorly structured PBL sessions may lead to superficial learning if students are not adequately guided [44]. Therefore, successful implementation requires well-defined learning objectives and strong instructor support to ensure that students engage meaningfully with the material. Another important note from this report is that simulation can be implemented in conjunction with other pedagogical approaches such as problem-based learning. It is interesting to note that scenario-based simulation typically has at its core a case around which learning activities are built. This might explain why both learning methods were practically combined in the reported cases. Furthermore, it shows that with an appropriate level of competence on the part of educators and creative adaptation of methods and pedagogies, simulation-based learning can be integrated into other established learning methods or pedagogical approaches [45]; [46].

### Integration of simulation-based training into medical curricula

The growing recognition of simulation-based learning has led to its increasing integration into medical curricula worldwide. Many medical schools have adopted simulation as a formal component of clinical training, reinforcing its role in competency-based medical education [31]. Research indicates that simulation-based training improves student confidence, enhances patient safety, and provides opportunities for deliberate practice in a risk-free environment [47]. However, challenges remain in fully integrating simulation into curricula, particularly concerning resource allocation, faculty training, and assessment strategies. Implementing high-fidelity simulation requires significant investment in equipment, infrastructure, and instructor development, which may not be feasible for all institutions [48]. Additionally, there is an ongoing need to refine assessment methods to accurately evaluate simulation-based competencies and ensure that learning outcomes align with real-world clinical expectations.

Curricular integration is important in regard to simulation. This is very important for emphasis as most reports did not highlight their strategies and specific ways of ensuring that simulation was effectively integrated into the curriculum to standardize it as pedagogical practice. This, however, is important for consistency and sustainability of practices. This is also important in order to ensure that the resources, time, and efforts committed to simulation-based medical education can be matched to program-associated competencies. By extension, the goals or objectives of specific simulation activities can be properly built on the programme related competencies and milestones in this regard. Considerations regarding proper curricular integration will also address the most suitable modalities and the level of fidelity that would be required in efforts to optimize the impacts and outcomes of simulation activities [49]. ​ Just as Moro and Gregory [50] highlighted opportunities in the use of visualizations to enhance face-to-face learning in medical and biomedical sciences, it is equally important to properly consider various contexts—such as online learning, face-to-face instruction, virtual environments, laboratories, or didactic settings—when promoting and utilizing simulation. Furthermore, empirical data and evidence of educational impacts will be needed to validate the practical applications of technologies and innovative approaches to the use of simulations in biomedical science education.

#### Recommendations

1. Educators and academic leaders in biomedical sciences should consider the importance of simulation-based medical education for learners.
2. To effectively use a simulation-based medical education approach, there is a need to acquire the triad of pedagogical, technological, and content competencies. Therefore, educators should develop technical competencies to effectively facilitate learning with simulation.
3. Educators who choose to use simulation-based medical education should also consider the curricular integration of this approach for sustainability and optimal benefits. In addition to pedagogical practices and assessment outcomes, reports concerning such efforts should include initiatives toward curricular considerations and integration.
4. The prospects of simulation-based medical education and the opportunities therein should be further explored by educators.
5. Since reports indicate that diverse disciplines have successfully developed simulation-based medical education, institutions should encourage all relevant disciplines to explore this approach to teaching and learning.
6. Adequate physical infrastructure and technological resources or support should be provided to support the deployment of simulation in terms of types and fidelity for optimal outcomes.

## Limitations

This scoping review could not include a meta-analysis on simulation-based medical education due to the significant heterogeneity of the data and information obtainable using the methods of review, and from existing literature. Also, the review was limited to approximately ten years, so it could not comprehensively cover the history and evolution of simulation-based medical education. Restricting the included articles to those published in English is also another limitation.

## Conclusion

Simulation-based and technology-enhanced learning has revolutionized medical education by providing experiential learning opportunities, improving skill acquisition, and fostering student engagement. While simulation enhances competency and knowledge retention, its effectiveness is maximized when integrated with traditional teaching methods rather than used as a standalone approach. Emerging technologies such as VR and AR offer promising educational advantages but should be implemented with careful consideration of cognitive load and accessibility. Moving forward, further research is needed to explore best practices for integrating simulation into medical curricula, ensuring that it remains an effective and sustainable component of medical education.

## Data Availability

All data generated or analysed during this review are included in this published article and its supplementary information such as the search strategy. As this is a scoping review, the sources of data comprise publicly available literature identified through systematic database searches. A detailed list of included studies, along with the search strategy and data extraction forms, has been provided. Further information is available from the corresponding author upon reasonable request.

## Appendix

**Table 3:** Appendix I: Search strategy- PubMed

Search Strategy for the primary search in PubMed	
(("computer simulation"[MeSH Terms] OR ("computer"[All Fields] AND"simulation"[All Fields]) OR"computer simulation"[All Fields] OR"simulation"[All Fields] OR"simul"[All Fields] OR"simulate"[All Fields] OR"simulated"[All Fields] OR"simulates"[All Fields] OR"simulating"[All Fields] OR"simulation s"[All Fields] OR"simulational"[All Fields] OR"simulations"[All Fields] OR"simulative"[All Fields] OR"simulator"[All Fields] OR"simulator s"[All Fields] OR"simulators"[All Fields]) AND ("based"[All Fields] OR"basing"[All Fields]) AND ("instruct"[All Fields] OR"instructed"[All Fields] OR"instructing"[All Fields] OR"instructional"[All Fields] OR"instructions"[All Fields] OR"instructive"[All Fields] OR"instructively"[All Fields] OR"instructiveness"[All Fields] OR"instructs"[All Fields] OR"teaching"[MeSH Terms] OR"teaching"[All Fields] OR"instruction"[All Fields]) AND ("students, medical"[MeSH Terms] OR ("students"[All Fields] AND"medical"[All Fields]) OR"medical students"[All Fields] OR ("medical"[All Fields] AND"students"[All Fields]))) OR (("undergraduate"[All Fields] OR"undergraduate s"[All Fields] OR"undergraduated"[All Fields] OR"undergraduates"[All Fields]) AND ("student s"[All Fields] OR"students"[MeSH Terms] OR"students"[All Fields] OR"student"[All Fields] OR"students s"[All Fields]) AND"post"[All Fields] AND ("sars cov 2"[MeSH Terms] OR"sars cov 2"[All Fields] OR"covid"[All Fields] OR"covid 19"[MeSH Terms] OR"covid 19"[All Fields]) AND ("pandemic s"[All Fields] OR"pandemically"[All Fields] OR"pandemicity"[All Fields] OR"pandemics"[MeSH Terms] OR"pandemics"[All Fields] OR"pandemic"[All Fields]))	
